# In a Coal Mine with Miners

**Published:** 1889-03-09

**Authors:** 


(*To be continued.*)

### In a Coal Mine with Miners.

By a New Hand.

"See everything once, my boy," was the parting injunction of my father on launching his son on that sea of trouble called the World. The injunction was wise—others who have followed it will agree in this statement—and one father of my acquaintance has improved upon it. The improvement consisted in the practical application of the principle by a personally-conducted tour, in his son's company, through all the haunts of amusement, vice, and temptation which the young Woolwich cadet would find in the great metropolis of London. Widely sympathising with the poor, my father's injunction has been followed by his son, who has visited and personally inspected the dwellings of the poor and the artisan at work, in many counties and at various trades. No one ought to aspire to be a legislator, or to attempt guide the helm of State, unless or until he has tried to see everything once, and so, in a measure, to possess the means of forming a wise conclusion on the varied and many-sided aspects of human life and labour, which legislation has to regulate in this our day. Acting on this principle, the writer has devoted many years of his life to its practical application, and, in this spirit, he has to-day been in a coal mine with the miners at their work. The mine is the property of the Grassmoor Company (Limited), in Derbyshire, and was until recently under the immediate management of Mr. Winter Barnes, a rising young engineer of great promise and no mean experience.

### Above and Below the Earth's Surface.

Arrived at the office of the Grassmoor Company, the first impression conveyed to the visitor is one of admiration at the self-contained character of all the arrangements. The workers may be roughly divided into those who remain at the mouth and those who labour in the mine itself. The former work for twelve hours a-day (from six a.m. to six p.m.), with an interval for meals, whilst the latter work for eight hours, *i e.*, from six a.m. to three p.m., exclusive of the additional hour it takes to enter and leave the mine, with an interval of a quarter of an hour only for a meal at 10.30 a.m. Men and boys work the same hours, and the labour throughout is very exacting and full of fatigue. It has been said that the arrangements are such as to make the mine self-contained. In explanation, it may be stated that everything required to equip and work the mine is made on the premises. Bricks, boxes or coal carriages, railway waggons, tools, implements—everything, in fact, except iron rails and the more complicated portions of the machinery, are made by the workers at the mouth of the pit. What is done below will be explained later on. It may be added, however, that nothing the mine produces is wasted. Its products consist of coal, and what is locally known as "dirt," that is, of small, loose stones and clay. The coal proper, or large coal, is raised to the surface and placed at once in the railway trucks, ready for transit to the metropolis. The small coal, or dust, is passed on by machinery to a row of furnaces, where it is speedily converted into coke. The dirt also consists of two portions, large and small. The former is built up at the back of the miners, where it supports the roof, and to the level of which the tops of the "gates" or small roadways fall, whilst the small dirt, or shale or clay, is sent to the surface, where it is ground to dust, and afterwards made into bricks. The miners, however, are only paid by results obtained in coal, *i.e.*, by so much per ton of coal raised.

### The Shaft or Entrance.

The shaft is the mouth of the mine, down which the workers are conveyed to their labours, and up which the coals are brought. It has been forcibly remarked by someone, somewhere, that of all horrors, ignorant fear creates the greatest and the worst. The unknown is always a cause of dread, and the sensation experienced by the novice on descending the shaft of a mine for the first time certainly gives the lie to Virgil, as it goes far to prove the fallacy of *Facilis descensus Averni*. In truth, the *descensus* is most uncomfortable, and, so far from being easy, it is decidedly disagreeable. Some, if not all, must be familiar with the sensation resulting from that worst of all dreams—the feeling of being dropped suddenly from a great height. Those who can remember the feeling will readily realise some of the sensations experienced on going down the shaft. The heart's action is greatly accelerated, the breathing is rendered difficult, the head swims, and the lungs are almost emptied of air. Indeed, and for these reasons, whereas the cage is worked at a speed of thirty miles an hour when it contains minerals only; when men are its burden, then a rate of fifteen miles is the utmost a strong man can bear, without injury or danger, during a descent of a quarter of a mile in length. The ascent, however, is less exacting and unpleasant, but the reason for this it is not easy to explain. Practice, however, makes people quite indifferent to such sensations, but the effect upon a boy of twelve, when he descends a mine for the first time, to commence a life of toil in the bowels of the earth, may well be imagined and sympathised with.

### An Underground Railway.

Arrived at the bottom of the shaft, we alight in a dark but spacious chamber, resembling nothing so much, perhaps, as Baker Street Station on the Underground Railway, except that the atmosphere of the mine is far better than that of the station. Lit by gas, with an arched brick roof, the floor being traversed in all directions by iron rails, on which are many small trucks filled and empty; the constant noise made by the carriages in motion; the bustle, the gloom, and the generally dirty aspect of everything, all combine to emphasise this impression. Boys and men are busy receiving the full trucks as they arrive from the furthermost workings of the mine, or in detaching the empty waggons as they are brought down from the top after having deposited their load in the railway trucks above. All is bustle—continuous and sustained labour. There is, however, no appearance of hurry, as each employé seems to know his duty, and to stick to it as if he were working for himself instead of for another.

### The Mine Proper.

The office of the deputy, or foreman of the mine, is first visited, where our lamps are lit, and we then proceed to examine the workings. Leaving the station at the bottom of the shaft, the works are approached first by roads, along the centre of which iron rails for the trucks are placed. At intervals on either side of the roads are the gates or entrances which lead by narrow passages between the "goafs" or "gobs," or exhausted workings, to the face of the "bank," or coal seam proper, where the miners are at work. The coal seam or bank, which is technically known as the face, shows in section first a stratum of rock at the top; then what is called dirt or shale, as already described; then the coal seam proper, which varies in width; then some more dirt; then a further coal seam; and, last of all, the bind or clay, which is taken to the surface and largely used in brickmaking. It can be imagined that the amount of dirt exercises considerable influence over the amount of wages a collier is able to earn. In some mines the coal seam is extensive, and the quantity of dirt small. This is the case in the North of England, where the seam is always larger than in Derbyshire. Here the quantity of dirt is regarded by the Yorkshire miners as so great as to render it impossible for them to work under such conditions. Nature, however, makes compensation to the Derbyshire miners, for the risk to life, owing to the comparatively small quantity of gas generated in a mine where the coal seam is relatively narrow, is so small as to make accidents as uncommon as they are easy to avoid; whereas, with the larger seams found in the Yorkshire mines, the risks to life are greatly heightened, and it is there that the worst accidents occur. The temperature of the Derbyshire mines is far lower than that of those in Yorkshire, and the conditions under which the men work are therefore better in the former collieries than in those of the latter. It seems at first sight remarkable that any man should, of his own choice, become a miner. As a matter of fact, mining, like other occupations, has its fascinations for those engaged in it, and a good workman becomes greatly interested in all that concerns the working of the mine, and, according to his lights, persuades himself that he has one of the most enjoyable of occupations. Many merry groups may be encountered as the men knock off work for a quarter of an hour to take what they call their "snap." This snap is to be seen suspended in little calico bags from the roof of the gates to avoid its being consumed by the mice which are to be found in considerable numbers wherever the works are proceeding.

### Administration and Staff.

The whole organisation of the mine—its workings, ventilation, and management are in charge of a mining engineer. Under him are two deputies, one in charge of the day shift from six a.m. to three p.m., and the other in charge of the night shift from nine p.m. to six a.m. The night deputy is assisted by two, and the day deputy by four firemen, whose duty it is to move about amongst the men and works, and see to the condition of the ventilation, the new workings, and the general discipline in that portion of the mine of which they have charge, the deputy being held responsible for the whole. At the Grassmoor Mine there were 70 boys and 390 men on the day shift, and 9 boys and 27 men on the night shift. To enable the work to proceed, a staff of ponies and horses is employed to move the boxes or trucks to and from the workings and the bottom of the shaft, sixty-seven being maintained in the stables in the main pit.

### Stables, Horses, and Ponies.

These stables are spacious and lofty, being some fourteen feet high, in consequence of the altitude at which the strong strata was found, and which it was necessary to reach in order to avoid constructing arches to carry the roof. Attached to the stables are three hospitals, in which the invalided horses are treated when suffering from bronchitis, a prevalent disease, due to the great difference in temperature between the gates and the roads, especially in winter. Horses and ponies live in a mine for many years, and apparently flourish, their coats being usually in fine condition, owing to the effect which the coal has upon them. It is a remarkable sight to meet a row of trucks drawn by horses or ponies in a narrow gate, the altitude of which is barely sufficient in places to enable the animals to keep their heads at a normal height. Despite this, they move along at a brisk pace, and seem to be absolutely indifferent to their surroundings and to the risks which attach to them. The men and boys become very much attached to the horses and ponies, and this feeling is reciprocated by the animals, who will go through a variety of antics and perform astounding tricks at the instance of their immediate attendants. Cases of cruelty or ill-treatment are reported to be rare, and the sympathy existing between the horses and ponies and their drivers, constitutes one of the brightest incidents in mining life. Near the stables, though at a lower level, is placed a huge furnace, which is kept going night and day, in order to secure the proper ventilation of the mine. The roads, stables, and station at the bottom of the shaft are all lit by gas, which is made on the surface and transmitted with the aid of a steam-jet to a receiver at the bottom of the mine.

